# Role of Histone Post-Translational Modifications in Inflammatory Diseases

**DOI:** 10.3389/fimmu.2022.852272

**Published:** 2022-02-24

**Authors:** Yingying Lin, Ting Qiu, Guifeng Wei, Yueyue Que, Wenxin Wang, Yichao Kong, Tian Xie, Xiabin Chen

**Affiliations:** ^1^ School of Pharmacy, Hangzhou Normal University, Hangzhou, China; ^2^ Key Laboratory of Elemene Class Anti-Cancer Chinese Medicines, Engineering Laboratory of Development and Application of Traditional Chinese Medicines, Collaborative Innovation Center of Traditional Chinese Medicines of Zhejiang Province, Hangzhou Normal University, Hangzhou, China; ^3^ Department of Pharmacology, School of Pharmacy, Nanjing University of Chinese Medicine, Nanjing, China

**Keywords:** inflammation, immune, epigenetic, inflammatory diseases, histone modifications

## Abstract

Inflammation is a defensive reaction for external stimuli to the human body and generally accompanied by immune responses, which is associated with multiple diseases such as atherosclerosis, type 2 diabetes, Alzheimer’s disease, psoriasis, asthma, chronic lung diseases, inflammatory bowel disease, and multiple virus-associated diseases. Epigenetic mechanisms have been demonstrated to play a key role in the regulation of inflammation. Common epigenetic regulations are DNA methylation, histone modifications, and non-coding RNA expression; among these, histone modifications embrace various post-modifications including acetylation, methylation, phosphorylation, ubiquitination, and ADP ribosylation. This review focuses on the significant role of histone modifications in the progression of inflammatory diseases, providing the potential target for clinical therapy of inflammation-associated diseases.

## Introduction

Inflammation is a response of the immune system to harmful stimuli including but not limited to microbial stimuli, pathogens, traumatic stimuli, toxic stimuli, or post-ischemic stimuli (1). It is a frequent occurrence to protect organisms from the spread of infection and recover the affected compartments to a normal state, which is called “resolution” (2). However, if the inflammation fails to subside, it will turn to a trouble itself and contribute to the pathogenesis of a great sort of inflammatory diseases (3).

The study of epigenetics, especially histone modifications, and their involvement in inflammation disease is still an emerging research filed, but it is becoming to attract more and more attention and growing at a fast pace. As the basic proteins in both eukaryotic and prokaryotic cells, histones combined with DNA constitute the nucleosome structure (4). Post-translational modifications (PTMs) of proteins are rising as a pivotal means by which chromatin could be altered to regulate gene expression; in other words, intracellular metabolites can modulate immunity. PTMs of histones on both the histone core and N-terminal tails affect a variety of biological processes such as transcription, replication, and chromosome maintenance (5).

**Figure 1:**
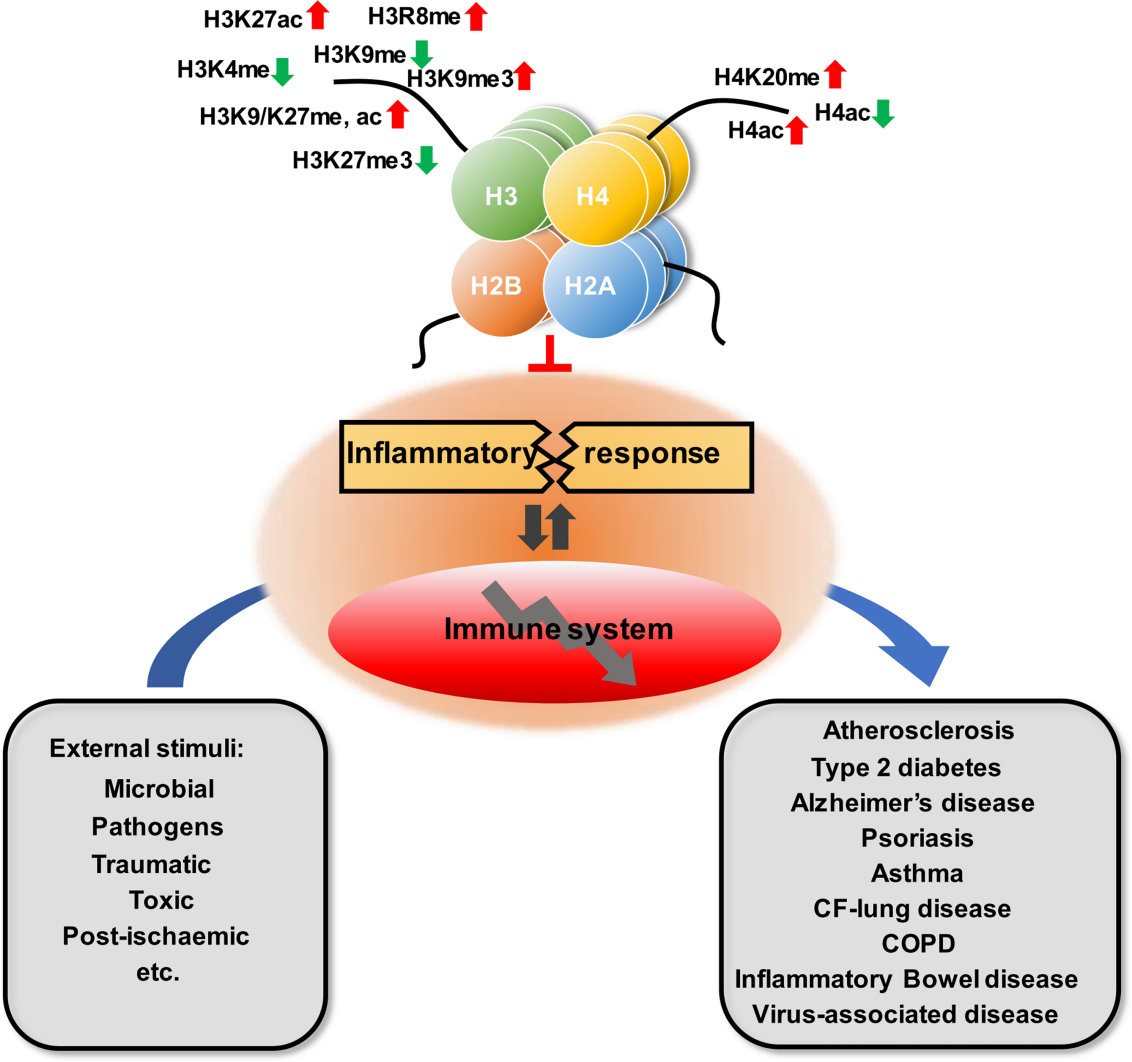
Overview of the role of histone modification in inflammatory disease.

Related studies have shown that the modifications of histones are closely associated with the occurrence of inflammatory diseases, including atherosclerosis, type 2 diabetes, Alzheimer’s disease, psoriasis, asthma, multiple chronic lung diseases, and inflammatory bowel disease (**Table 1** and **Figure 1**). This article offers an illustrative landscape of histone modifications as significant regulators for chronic or virus-related inflammatory diseases, providing a perspective on the therapy of inflammatory diseases.

**Table 1:** Histone modifications in inflammatory diseases.

Diseases	Target	Modification	Regulators	Reference
Atherosclerosis	HDAC1	Deacetylation	KLF5, miR-224-3p	(6, 7)
	HDAC3	Deacetylation	miR-19b	(8)
	HDAC4	Deacetylation	miR-200b-3p	(9)
	HDAC6, H3K9	Deacetylation	NORAD	(10)
	SIRT6	Deacetylation		(11)
	H3K9, H3K27	Methylation↓		(12)
	H3K27	Trimethylation↓	EZH2	(13)
Type 2 diabetes	H3K9, H2AK119,	Dimethylation, Acetylation, Ubiquitination↑	DBP	(14)
	H2BK120	Trimethylation↑	GLUT4	(15)
Alzheimer’s disease	H3K9	Deacetylation	Keap1	(16)
	HDAC3	Methylation, Acetylation↑	amyloid-β42	(17)
	H3K9, H3K27			
	SIRT1, 3, 6	Deacetylation↓		(18)
	H3K4, H3K9	Trimethylation		(19)
Psoriasis	H3, H4	Acetylation↓		(20)
	H3K4	Methylation↑		
	H3K27	Trimethylation↑	EZH2, JMJD3	(21, 22)
	H3K27	Acetylation↑		(23)
Asthma	H3K9, K14, K18, K23, K27, K36, H2B1KK120, B2BK20, BK16, BK20, BK108ac, BK116ac, BK120ac	Acetylation↑		(24)
	H2BK5, H2BK11	Acetylation↓		
	H3	Citrullination↑		(25)
	HDAC4	Deacetylation↑	Slug, CXCL12	(26)
Chronic obstructive pulmonary disease	H3K4, H3K27	Methylation, Acetylation↑	IL6-AS1	(27)
	H3K9	Trimethylation↓		(28)
	HDAC2	Deacetylation↓		(29)
	SIRT1	Deacetylation↓		(30)
Cystic fibrosis lung disease	HDAC6	Deacetylation		(31)
	HDAC7	Deacetylation		(32)
Inflammatory bowel disease	H3K27	Trimethylation	EZH2	(33)
	H3K4	Trimethylation↑		(34)
	H4K20	Monomethylation	SETD8	(35)
	H3R8	Methylation↑	PRMT2	(36)
	H3K27	Acetylation		(37)
	H1, H3	Citrullination		(38)
	SIRT1	Deacetylation↓		(39)
Virus-associated disease				
SV40	H3/H4	Hyperacetylate	P300/CBP	(40, 41)
	H3K9/H4K20	Methylation		(42)
Merkel cell polymavirus	H3K27	Trimethylation↓		(43)
	H3K27	Acetylation	P300/CBP	(44)
HPV	H3	Acetylation↓	E6 and p300/CBP, TIP60, HDAC1, HDAC2	(45, 46)
	H3	Acetylation↑	E7 and p300/CBP	(44)
	H3	Methylation ↑	E6/E7 and EZH2	(47)
	H3	Methylation↓	E6/E7 and KDM6A/KDM6B	(48)
HBV	H3/H4	Acetylation	HBx and p300/CBP, HDAC1, SIRT1	(49, 50)
	H3K4/H3K9	Methylation	HBx and SETDB1, EZH2, SMYD3,	(51, 52)
HDV	H3	Acetylation		(53)
HCV	H3/H4	Acetylation	H2AX	(54)
	H3	Methylation	KDM5B, LSD1, G9a, EZH2	(55, 56)
HIV	H3	Acetylation	CTIP2, HDAC1/2, BRD4	(57, 58)
	H3	Methylation	LSD1, SET1, EZH2	(57, 59)
SARS-CoV-2	H3	Acetylation	NSP5 and HDAC2, SIRT1	(60, 61)
	H3K9	Methylation		
	H3	Citrullination↑	PAD4	(62–64)

Symbol ↑ represents upregulated，symbol ↓ represents downregulated.

## Immunity and Inflammation to Inflammatory Disease

Inflammation is a coordinated immune response to tissue injury and pathogenic or non-pathogenic infections. The process involves a variety of cell responses, including immune cell migration and cytokine release (65). The function of inflammation is to repair the damage or resist the infection and restore the balanced state through redeploying the immune system (66). In the acute phase of inflammation, immune cells migrate to the injury site to contain the infection and repair damaged tissue, and then the lesion begins to heal. Effective, rapid, and targeted resolution is the ideal inflammatory response. Persistent inflammatory response can give rise to serious consequences, such as tissue damage, immune dysfunction, organ lesion, cancer, and even death, finally resulting in chronic diseases (67–70). The inflammatory process involves complex regulations of target genes through mass of signalings or epigenetic mechanisms.

### Histone Post-Translational Modifications

**Figure 2:**
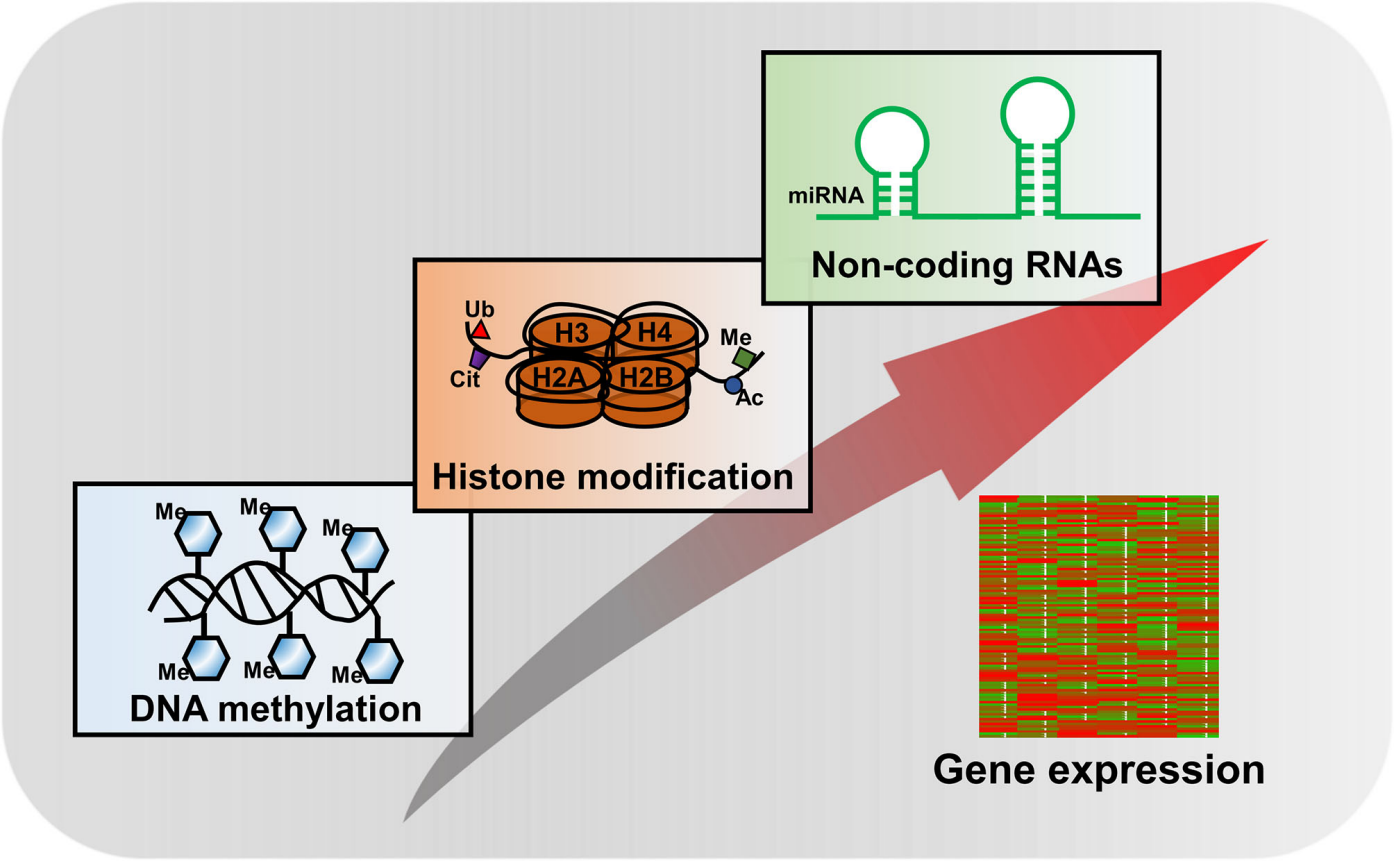
Major epigenetic mechanisms in regulation of gene expression.

Histone modifications are one of the key components of epigenetic mechanisms, producing heritable changes in gene expression without alterations in the DNA sequence (**Figure 2**). Over the past two decades, explosive discoveries of epigenetics have been laid down to unravel the mysteries of various biological processes.

The nucleosome core is formed by a histone octamer consisting of two copies of H2A, H2B, H3, and H4 and surrounded by ~150 base pairs of DNA, which is the basic functional unit of chromatin (71). PTMs of histones mostly present on the amino-terminal tail domains of the histones, but yet new data showed it also happened on the core of histones which could alter chromatin architecture by directly mediating the protein–DNA interaction (72). Besides the long-studied PTMs such as acetylation, methylation, ubiquitination, phosphorylation, citrullination, glycosylation, formylation, deamination, ADP ribosylation, proline isomerization, and sumoylation (4), a series of novel PTMs were identified recently, including crotonylation (73), propionylation, butyrylation (74), and lactylation (75). Among these, histone acetylation and methylation are the best understood PTMs.

Histone acetylation is mostly related to active transcription, while histone methylation is a kind of complex which depends on the specific methylated sites to regulate transcriptional states. The coexistence of mono-methylated H3K4 (H3K4me1) and acetylated H3K27 (H3K27ac) is the hallmark of active enhancers (76, 77). Histone methylation is commonly mediated by histone methyltransferases (HMTs), which specifically occurs on histone H3 and H4 at distinct lysine or arginine residues (78). Moreover, the enzyme family of the Complex of Proteins Associated with Set1 (COMPASS) is indispensable for H3K4 methylation (79, 80). Normally, histone methylation is considered to be a stable epigenetic mark because it sustains a stable overall charge of the histone tails, while with increasing level of methylation, it will lead to the increase of basicity, hydrophobicity, and affinity for DNA and then alter chromatin and regulate gene transcription (78).

**Figure 3:**
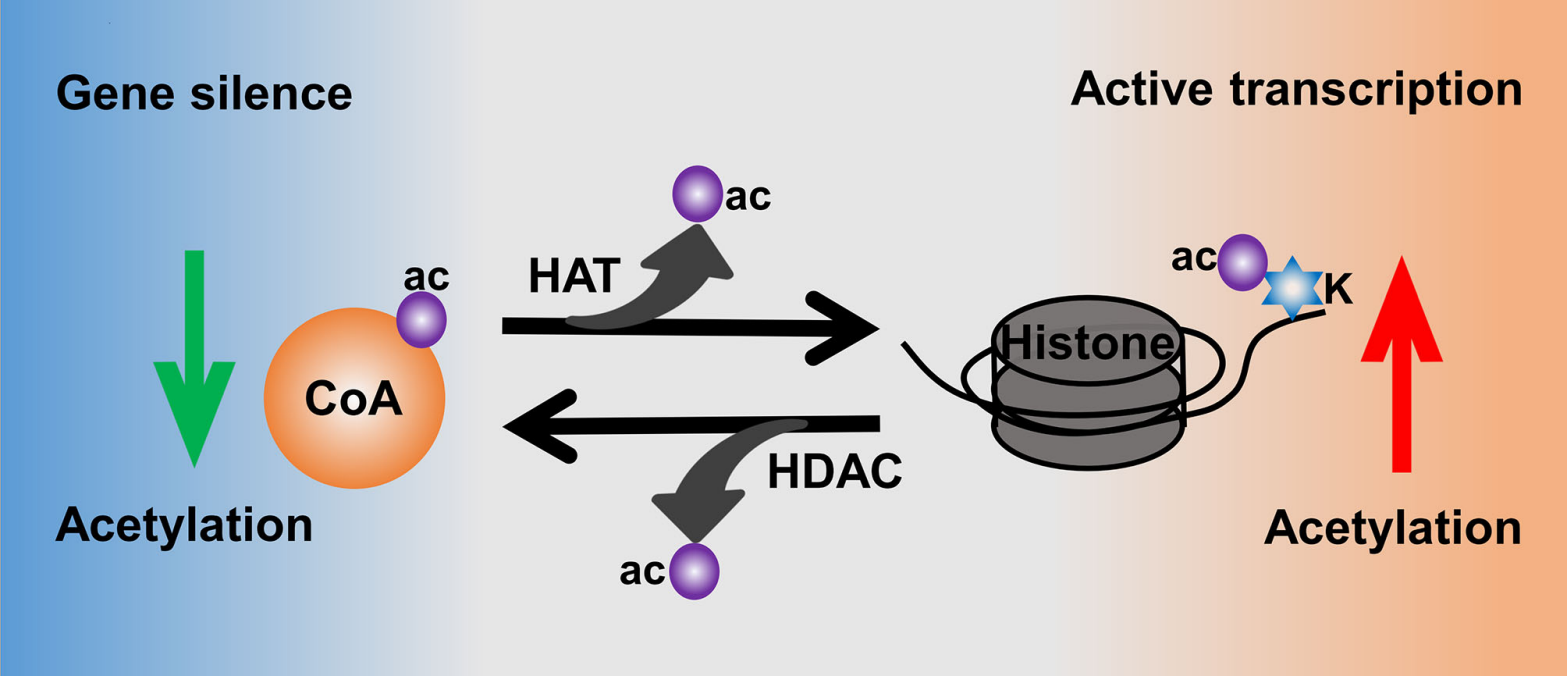
Histone acetylation mediated by the activities of HATs and HDACs.

Moreover, histone acetylation is highly mediated by the activities of histone acetyltransferases (HATs) and histone deacetylases (HDACs) (81) (**Figure 3**). HATs catalyze the transfer of acetyl from donor-acetyl coenzyme A to lysine residues of the histone peptide, which could increase the level of histone acetylation to make the chromatin sustain an active transcription (78). In contrast, HDACs are responsible for catalyzing the removal of acetyl from ϵ-amino groups of conserved lysine residues at the histone amino terminal tail, thus leading to a low level of acetylation as well as heterochromatin and gene silence (82, 83). There are 18 human HDACs identified and categorized into four classes: class I are Rpd3-like proteins and composed of HDAC1, HDAC2, HDAC3, and HDAC8; class II are Hda1-like proteins consisting of HDAC4~HDAC7 and HDAC9~10; class III are Sir2-like proteins including SIRT1~7; and class IV only contains HDAC11 (84).

Otherwise, the polycomb group consists of PRC1 and PRC2 protein complexes maintaining catalytic properties for distinct histone modifications (85). As an E3 ubiquitin ligase, PRC1 mediated histone H2A mono-ubiquitylation for gene silencing (86, 87), whereas PRC2 compacts chromatin and catalyzes the methylation of histone H3K27 *via* its catalytic subunit EZH1/2 (88). Furthermore, additional enzymes related to histone modifications include the phosphorylation of specific serine groups by histone kinases (HKs) (89), catalyzing the conversion of butyryl-CoA to crotonyl-CoA by acyl-CoA dehydrogenase, ACADS, acyl-CoA oxidase, and ACOX3 (90); the attachment of ubiquitin (Ub) by PRC1 (91); the ADP-ribosylation of histones by poly(ADP-ribose) (PAR) units (92); and sumoylation by small ubiquitin-like modifiers (SUMOs) (93). Although plenty of histone modifications has been identified, the hidden mechanisms for epigenetic regulation still have a long way to go.

## Histone Modifications in Inflammatory Diseases

### Atherosclerosis

Atherosclerosis (AS) is characterized by large and medium arteries, which is caused by chronic inflammatory disorder of the arterial vessel wall, and it is commonly considered as a major contributor of cardiovascular diseases (CVDs) including stroke and myocardial infarction (94, 95). Monocyte-derived macrophages mainly contribute to the development of AS (96, 97). Accumulating research showed the significant effects of histone modifications in the progression of AS. Therein, HDACs play a non-negligible role (98). HDAC1 was targeted by microRNA (miR)-410 to increase the level of IKBα through suppression of NF-κB by KLF5, thus preventing the development of AS (6). Meanwhile, overexpression of HDAC1 suggested to promote the anti-AS effects of miR-224-3p-mediated FOSL2 inhibition *via* HIF1α deacetylation (7). The defect of myeloid HDAC2 in high-calorie diet-fed (HFD) LDLR^-/-^ male mice leads to a significant AS reduction without affecting plasma lipid and lipoprotein profiles. The inhibition of HDAC2 prevents monocytes and macrophages from nutrient stress-induced dysfunction and reprogramming which could transform into atherosclerosis (99). Excessive HDAC3 prevents inflammation to restrain atherosclerosis progression through inactivating NF-κB/p65 *via* upregulation of PPARγ, and it was mediated by miR-19b (8). Epicardial adipose tissue (EAT) may target HDAC4 and cause endothelial cell damage induced by miR-200b-3p, promoting oxidative stress (9). LncRNA non-coding RNA activated by DNA damage (NORAD) is a novel long non-coding RNA (lncRNA); it could recruit HDAC6 to enhance H3K9 deacetylation and consequently suppress the transcription of the VFGF gene which was supposed to enhance vascular endothelial cell injury and atherosclerosis (10). Endothelial–mesenchymal transition (EndMT) is associated with atherosclerosis and plaque instability. Both *in vivo* and *ex vivo* knockout of Hdac9 could prevent EndMT through sustained endothelial protein expression and alleviate the increase in mesenchymal proteins, thus slowing the development of atherosclerosis (100). HDAC9 is also a regulator of atherosclerosis plaque stability and IKK activation to drive inflammatory responses in both endothelial and macrophages cells (101). A lower expression of Sirtuin 1 (SIRT1) leads to ovariectomy (OVX)-induced arterial senescence and atherosclerosis in apolipoprotein E-knockout (ApoE-KO) mice (102). SIRT3 has been reviewed to play multiple roles in the development and regression of atherosclerosis (103). The expression of SIRT6 is reduced in both human and mouse plaque of vascular smooth muscle cells (VSMCs), and it is indicated to regulate telomere maintenance and VSMC lifespan as well as inhibit atherosclerosis depending on its deacetylase activity (11).

In addition to histone acetylation, histone methylation and citrullination also play a decisive role in atherosclerosis progression. Methylation of H3K9 and H3K27 was decreased in atherosclerosis plaques in smooth muscle cells (SMCs), and H3K4 methylation showed a significant association with the severity of atherosclerosis (12). Besides, histone H3K27 trimethylation could be catalyzed by PRC2 with EZH2, which is deemed to increase macrophage inflammatory responses (13, 104). A recent study showed that *Ezh2*-deficient mice reduced the levels of H3K27me3 and decreased H3K27 methyltransferase activity and also showed a significant reduction of lesion size suggesting the improvement of atherosclerosis (105). Citrullinated histones (Cit-histones) are associated with neutrophil extracellular trap (NET) release and are involved in different AS events *in vitro*. Cit-histones were pro-atherogenic mediators which can accelerate low-density lipoprotein aggregation when it is released at the lesion, thus slowing down atherosclerosis progression (106).

### Type 2 Diabetes

Over the years, type 2 diabetes (T2D) becomes a worldwide disease with a leading prevalence of incidence. Unhealthy diets, physical inactivity, and aging are the major risks of T2D, which can lead to impaired insulin action and secretion (107). It has long been uncovered that chronic inflammatory processes and epigenetic mechanisms were underlying in the pathogenesis of T2D (108–110). Recently, increasing data have focused on the role of histone modifications in T2D progression. Increasing levels of histone modifications such as H3K9me2, H3K9Ac, H2AK119Ub, and H2BK120Ub in the heart of T2D rats can be attenuated by telmisartan, which could ameliorate T2D cardiomyopathy (14). Besides, exendin-4 induced a reversal of pancreatic histone H3K9 and H3K23 acetylation, and H3K4 mono-methylation and H3K9 di-methylation might improve T2D progression (111). Increased H3K9me3 in the Slc2a4 promoter could reduce the expression of GLUT4, encoded by Slc2a4, which contributes to glycemic impairment in T2D (15). Acetylation of H3K9 at the promoter region of clock gene Dbp and DBP mRNA expression in omental adipose tissue, a compartment related to the mechanism of T2D, was significantly lower in T2D patients (112).

Inhibition of HDACs has been frequently reported to attenuate T2D progression. Reduction of pancreatic β cell mass is a hallmark of T2D which can alter insulin signaling. Moreover, reducing the expression of HDAC6 in pancreatic β cells tends to downregulate insulin signaling (113). Selective inhibition of HDAC3 by RGFP966 enhanced insulin secretion and synthesis which might retard the development of T2D (114). In addition, inhibition of HDAC3 accelerates vascular endothelial proliferation during vascular impairment caused by T2D through activating Nrf2 signaling by inhibiting Keap1 synthesis and Nrf2–Nox4 association, which indicated the potential of HDAC3 as an epigenetic regulator in T2D-related vascular complications (16). Elevated levels of HDAC7 lead to β cell dysfunction and the defects of T2D (115). SIRT4 has been reported to deactivate AMPK signaling, which leads to insulin resistance responses including inflammation and oxidative stress and inhibits insulin secretion directly, ultimately producing T2B (116). Defect of SIRT6 was supposed to increase H3K9 and H3K56 acetylation and TXNIP expression, which is important in maintaining β cell function and viability (117).

### Alzheimer’s Disease

Alzheimer’s disease (AD) is a typical age-related neurodegenerative disease, which is induced by chronic neuroinflammation with increased microglia and astrocyte activation, leading to cognitive impairment and dementia (118–122). Accumulation of amyloid-β plaques and tau tangles are two representative pathological characterizations of AD. Recent research has shed light on the critical role of epigenetic regulations especially histone modifications in AD progression (123–125). Using genome-wide RNA-interference-based screening, Yuan et al. identified 59 genes that might regulate age-related behavioral deterioration including cognitive decline in aging *Caenorhabditis elegans* (126, 127). Moreover, a neuronal histone 3 lysine 9 methyltransferase is one of the 59 genes identified; it was found to increase with age in the frontal cortex and correlate positively with AD progression. Meanwhile, multi-omics integrated by transcriptomic, proteomic, and epigenomic analyses of the postmortem human brain with AD revealed that the histone acetyltransferases for H3K27ac and H3K9ac were upregulated at the mRNA level and enriched specific to AD tissues at the protein level (17). In the same study with a fly model of AD, increasing levels of H3K27ac and H3K9ac in genome-wide aggravated amyloid-β42-driven neurodegeneration are observed. In parallel, however, another epigenome-wide association study employing the H3K9ac mark in 669 human prefrontal cortices discerned tau protein burden but not amyloid-β affecting 5,990 out of 26,384 H3K9ac domains, which showed a greater effect on the AD-related brain epigenome (128). Sirtuins have long been discussed to exert multiple functions in brain aging and neurodegenerative diseases such as AD (129–131). The expressions of SIRT1, SIRT3, and SIRT6 in the hippocampus and saliva were 1.5- to 4.9-fold reduced in elderly AD patients compared to healthy individuals of corresponding ages (18). Several other studies also indicated that SIRT1, SIRT3, and SIRT6 were reduced in AD patients in both mRNA and protein levels (132–134).

HDAC inhibitors were deemed to be innovative agents for AD therapy as its involvement in neurodevelopment, memory formation, and cognitive processes (135, 136). HDAC6 has been indicated to regulate tau acetylation reversibly, and a HDAC6-dependent surveillance mechanism that inhibits toxic tau accumulation has been put forward (137). Choi et al. found a HDAC6 inhibitor, CKD-504, which could dramatically change the tau interactome to degrade pathological tau in amyloid plaques and neurofibrillary tangles of AD model mouse brains through the proteasomal pathway, in the end rescuing cognitive decline in AD model mice (138). Another HDAC6 inhibitor, MPToG211, can significantly reduce tau phosphorylation and aggregation, which cause ubiquitination of phosphorylated tau proteins through reducing the binding of Hsp90 and HDAC6. This inhibitory activity ameliorates learning and memory impairment in AD animal models (139).

Despite genome-wide changes of histone methylations being displayed in aging and cognitive functioning, the impact of the diverse arrays of histone methylation has not been deciphered even in AD (140, 141). H3K4me3 has been targeted to recover prefrontal cortex synaptic function and memory-related behaviors by compound WDR5-0103, a newly identified H3K4me3 inhibitor (142). Likewise, H3K9me3 highly occupied epigenomes involved in synaptic transmission, neuronal differentiation, and cell motility, to remodel heterochromatin condensation, leading to a downregulation of the synaptic pathology of sporadic AD (19).

### Psoriasis

Psoriasis is a recurrent and chronic inflammatory skin disease with multiple pathological features such as vascular hyperplasia, abnormal keratinocyte proliferation, and infiltration of inflammatory cells into the dermis and epidermis (143). Multiple factors including genetic susceptibility, environmental factors, and innate and adaptive immune responses were involved in psoriasis, making it a complex disease. However, the exact etiology remains largely unknown. In recent years, accumulating studies have linked epigenetic network imbalances with psoriasis, considering it as one of the major causative elements for the disease (144, 145). In this regard, we focus on the effect of histone modifications on psoriasis. An analysis of peripheral blood mononuclear cells isolated from both psoriasis patients before and after therapeutic drug administration and healthy individuals shows that the levels of acetylated H3 and H4 were reduced while the level of methylated H3K4 was increased (20). The expression of H3K27me3 and its trimethylation mediator, EZH2, was also increased in the epidermis of psoriatic lesional skin compared to the normal one. Moreover, knockdown of EZH2 caused an abnormal proliferation of keratinocytes which could be reversed by its target gene Kallikrein-8 (KLK8) (21). Another study identified grainyhead-like 2 (GRHL2) binding at the promoter region of target gene EDC, which might inhibit the recruitment of histone demethylase Jmjd3 to the EDC promoters and increase the level of H3K27me3 leading to the inhibition of keratinocyte differentiation (22). ChIP-seq with anti-H3K27Ac in psoriatic and healthy skin identified an overexpressed enrichment of H3K27Ac in psoriasis (23). HDAC1 was overexpressed in psoriasis patients while SIRT1 was decreased in the basal layer of psoriasis patients compared to healthy controls (146). Activation of SIRT1 by resveratrol induced human keratinocyte damage through blocking the Akt pathway (147). The evidence linked histone modifications with psoriasis progression providing a therapeutic target for psoriasis.

### Asthma

Asthma is a common chronic inflammatory respiratory disease characterized by coughing, breath shortness, chest tightness, and wheezing, usually triggered by noxious agents or aeroallergens (148). Airway inflammation, hyperresponsiveness, and remodeling are the major contributors to the development of asthma (149). The role of epigenetic mechanisms in the pathophysiological process of asthma is progressively identified and confirmed (150–152). Using proteomics analysis of asthmatic lung tissues, Ren et al. identified 15 differentially modified acetylation sites, among which thirteen sites were upregulated including H3K9ac, H3K14ac, H3K18ac, H3K23ac, H3K27ac, H3K36ac, H2B1KK120ac, H2B2BK20ac, H2BK16ac, H2BK20ac, H2BK108ac, H2BK116ac, and H2BK120ac, while two sites were downregulated including H2BK5ac and H2BK11ac. These are potential acetylation sites related to asthma pathogenesis (24). The histone acetylation of orosomucoid 1 like protein 3 (ORMDL3) was mediated by histone acetylase p300 using a dual-luciferase reporter assay. p300 increased the mRNA levels of endogenous ORMDL3 by activating transcription from the ORMDL3 promoter. ORMDL3 expression and HAT activity were increased in the lung tissues of asthmatic mice. When p300 expression and HAT activity as well as aceH3 levels were impeded by C646, the expression of ORMDL3 would be reduced and relieve airway hyperreactivity, which improves airway inflammation and remodeling in asthma (153). With a genome-wide profiling of the enhancer-associated histone modification H3K27ac in bronchial epithelial cells (BECs) from asthma patients, 4,321 (FDR < 0.05) regions were identified to exhibit differential H3K27ac enrichment between individuals with or without asthma (154). Inhibiting H3K27me3 demethylation by a selective inhibitor GSK-J4 could improve the typical hallmarks of asthma, including airway inflammation, hyperresponsiveness, and remodeling, then alleviate the development of asthmatic disease (155). A study showed an elevated circulating H3cit level in stable asthmatics which is related to the enhanced lung extracellular traps (ETs) (25).

The activity of HDAC is distinctive in asthma progression (156). HAT and HDAC activities were associated inversely in blood monocytes isolated from healthy individuals and patients with asthma, and HAT activity was increased while HDAC activity was reduced during neutrophilic airway inflammation (157). HDAC1 protein expression was inhibited by intranasal curcumin to retard asthma severity in an allergic asthmatic mouse model (158). HDAC2 protein expression could be reduced by cigarette smoke exposure *via* enhancing AKT signaling, and it can be reversed by roxithromycin (RMX) treatment to increase HDAC2 expression and reduce airway inflammation (159). The expression of HDAC4 was upregulated in lung tissues of asthmatic mice, and it could deacetylate Kruppel-like factor 5 (KLF5) to upregulate Slug and CXC chemokine ligand-12 (CXCL12), thus triggering airway remodeling and promoting progression of asthma (26). Overexpression of SIRT6 reduced cell migration and proliferation, suppressed the activation of Smad3 phosphorylation induced by TGF-β1 treatment, and at the same time decreased the H3K9 acetylation level and the transcriptional activity of the c-Jun promoter (160). Moreover, SIRT1 restrained the inflammatory cytokine expression in primary bone marrow-derived macrophages (BMDMs) through the ERK/p38 MAPK pathways (161). The modulations suggested that upregulation of the expression of SIRT6 might improve airway remodeling in asthma.

### Chronic Obstructive Pulmonary Disease

Chronic obstructive pulmonary disease (COPD) is also a chronic inflammatory airway disease similar to asthma, involving an obstruction in airflow, which is reversible in asthma while being progressive and irreversible in COPD (162, 163). Aberrant neutrophilic inflammation is characterized by COPD, which contributes to airway damage and leads to alveoli loss, mucus production increase, and mucociliary dysfunction (164, 165). The underlying pathogenesis of COPD is still largely unknown. Existing research indicated that epigenetic mechanisms are associated with the disease progression in COPD (166). A novel lncRNA, interleukin 6 antisense RNA 1 (IL6-AS1) supposed to recruit early B-cell factor 1 to the IL-6 promoter to increase the H3K4 methylation and H3K27 acetylation, therein, increases airway inflammation in COPD (27). SUV39H1 is a histone methyltransferase; the levels of SUV39H1 and H3K9me3 were reduced in COPD patients. Reduction of SUV39H1 by administration of its specific inhibitor, chaetocin, or genetic knockdown, leads to a loss of H3K9me3 and enhances inflammatory responses in COPD (28). A proteome study of COPD and histone lysine crotonylation (Kcr) found 190 proteins upregulated and 151 proteins downregulated, among which 90 proteins were regulated by differentially expressed crotonylation sites and expressed differentially in COPD (167).

Targeting HDACs as a therapy approach for COPD has drawn more and more attention within recent scientific research (168). It has been shown that HDAC2 was downregulated in skeletal muscle of COPD patients (29). In addition, the HDAC2 protein level was decreased upon PM2.5 exposure, and myeloid-specific deficiency of HDAC2 enhanced PM2.5-induced M2 alveolar macrophage polarization which resulted in the progressiveness of COPD (169). Similarly, knockout of HDAC2 enhanced cigarette smoke (CS)-induced DNA damage, inflammatory response, and cellular senescence in mouse models, indicating that HDAC2 is the key player in CS-associated COPD disease (170). SIRT1 was shown to maintain a lower expression in CD28nullCD8 + T and NKT-like cells than in CD28+ cells from COPD patients and healthy controls, which was related to increased IFNγ and TNFα production, steroid resistance, and disease progression. Increasing expression of SIRT1 by treatment of multiple specific drugs such as prednisolone can reverse these activities as to reduce systemic inflammation in COPD (30).

### CF-Lung Disease (Cystic Fibrosis)

Cystic fibrosis (CF) lung disease is another life-threatening chronic inflammatory lung disease with various mutations in the cystic fibrosis transmembrane conductance regulator (CFTR), which assists in the regulation and clearance of mucus (171, 172). As a familial autosomal recessive disease, the related epigenetic mechanisms were gradually revealed (173). Increasing evidence showed that HDAC inhibitors could largely assist in correcting protein-misfolding diseases such as CF-related diseases (174). The most notable binding partner of HDAC6 is alpha-tubulin, which is reduced in CF cells. Inhibiting the expression of HDAC6 enables the restoration of tubulin acetylation to normal levels in CF cells (175) and reversal of the growth defects such as height and weight caused by CF (176). Depletion of HDAC6 in CF mouse models can also regain the growth and responsive activity to bacterial challenge and inflammatory phenotypes of wild-type mice (31, 177). Moreover, in HEK293 cells, inhibition of HDAC6 by suberoylanilide hydroxamic acid (SAHA) regulated both innate and adaptive immune responses of CF-lung disease-associated pathogenesis and progression (178). The majority of CF patients have a deletion of Phe 508 (△F508), which induces an efficient degradation of CFTR and then leads to premature lung failure (171). △F508 CFTR interacted with at least 638 proteins, which forms a △F508 CFTR interactome, and remodeling the interactome could promote the rescue of cystic fibrosis development (179). Hutt et al. revealed that HDAC7 was beneficial for restoring △F508 function through a SAHA-sensitive mechanism (32).

### Inflammatory Bowel Disease

Inflammatory bowel disease (IBD) is a chronic inflammatory disease with distinct gastrointestinal disorders, which maintains two common clinical forms including ulcerative colitis (UC) and Crohn’s disease (180). Not only genetic predisposition but also environmental factors and imbalance of intestinal bacterial flora tend to induce IBD incidence (181–183). As a multifactorial disease, the exact etiology of IBD still needs to be unraveled, but recent studies have linked the pathogeny of IBD with epigenetic mechanisms (184–186). H3K27me3 can be regulated by extracellular vesicles to control the differentiation of Th17 cells in ulcerative colitis, which plays a distinct role in the pathogenesis of IBD (187). Inhibiting the activity of EZH2 on H3K27me3 promoted the development of functional myeloid-derived suppressor cells (MDSCs), which was beneficial to promoting the anti-inflammatory effect to treat IBD (33). A large proportion of genes enriched in newly diagnosed pediatric IBD patients maintained a significant level of H3K4me3, which was related to the severity of intestinal inflammation during the progression of IBD (34). SETD8 is a typical histone H4K20 methyltransferase in which silence of SETD8 could significantly decrease the enrichment of H4K20me1 in the p62 promoter to regulate the expression of p62 and inhibit the inflammatory response in colitis (35). The level of H3K9 acetylation was decreased upon dextran sulfate sodium (DSS) treatment, which was reported to induce colitis by lessening the macrophage amount and the secreted inflammatory cytokines (188). Protein arginine methyltransferase 2 (PRMT2) presents a high expression in IBD patients; it could increase the asymmetric methylation of H3R8 at the promoter of the suppressor of cytokine signaling3 (SOCS3) to mediate colitis progression (36). A genome-wide profiling of H3K27ac of colon tissues from DSS-induced chronic colitis mouse model identified 56 candidate genes that are potentially involved in H3K27ac change, among which special typical enhancers were upregulated by H3K27ac that might be associated with the development of intestinal inflammation (37). Citrullination of histone H1 and H3 forms NETs to activate fibroblasts into myofibroblasts in triggering fibrosis, thus potentially alleviating IBD (38).

HDAC inhibitors (HDACi) were shown to play a positive role in the course of IBD (189–191). HDAC2, HDAC3, HDAC6, HDAC9, and HDAC10 have been reported to be especially associated with IBD (192). The family member IL-35 (EBI3/IL-12p35) was indicated to induce anti-inflammatory activity in UC (193), and the activity could be upregulated by histone acetylation *via* HDACi administration (194). SIRT1 displayed a lower activity in various IBD models, which is important for the generation of oxidative stress and the production of pro-inflammatory cytokines (39).

### Virus-Associated Diseases

Virus infections are closely correlated with many diseases, such as cancer and pneumonia. Inflammation induced by virus is the main driving force in disease development (195). Both virus life cycle and host cell viability are regulated by epigenetic modifications. After infection, the virus DNA was transported to the nucleus and then integrated into chromatin to form the virus genome nucleosomes (41). Although the composition of virus genome nucleosomes is simple, the epigenetic regulation especially histone modifications plays an important role in the expression of appropriate genes. Meanwhile, the infection also changes the epigenetic modifications in host cells, even resulting in the transformation of a normal cell to a cancer cell (41, 69, 196). Clarifying the changes in epigenetic modifications is increasingly needed in the therapy of virus-related diseases.

#### Polyomavirus

Polyomavirus infection can lead to chronic inflammation-associated diseases, such as cancer and nephropathy (197, 198). SV40 is a polyomavirus with a small double strand and circular DNA, which has been used as a model system to study basic aspects of histone modifications after virus infection. The viral DNA and histone were organized into a minichromosome, presented in virions and infected cells.

In the SV40 virions, H3 and H4 were hyperacetylated, along with the methylation of H3 on lysine9 (H3K9me1, H3K9me2, H3K9me3) and H4 on lysine 20 (H4K20me) (40, 199). Each methylated form of H4 was involved in different biological functions in cellular chromatin. H4K20me1 appears to associate with transcriptionally activation, while H4K20me2 is especially associated with DNA damage and repair (42, 200). It is consensus that H4K20me3 is concentrated in heterochromatin (201, 202). In the SV40 minichromosome, H4K20me1 appears to associate with transcription and may be necessary to compact minichromosome into the virion (40). The T-antigen in SV40 interacted with HAT and HDAC family members, such as p300, CREB-binding protein (CBP), HDAC1, and HDAC3 in host cells, resulting in the dysregulation of the genome (41). The T-antigen was associated with the inflammation responses (197). For the other polyomavirus, Merkel cell polyomavirus (MCPyV), the H3K27 trimethylation (H3K27me3) was repressed and the p300/CBP was upregulated (43, 203). The MCPyV sT was reported to interact with PP4 and the interaction interferes with the NF-kB pathway which was involved in the inflammation response (198).

#### Papillomavirus

HPV is a human papillomavirus with a circular, double-stranded DNA (204). High-risk HPV is the etiological factor of cervical carcinoma which is the fourth most common malignancy in women worldwide. HPV proteins are involved in the development of chronic inflammation which is the pivotal causal factor in the development of HPV carcinogenesis (205). It has been known that during HPV infection, certain epigenetic alterations occurred in HPV and host cellular genomes (206).

The main oncoproteins E5, E6, and E7 in HPV play important roles in viral life cycle and cancer development (207). The E5, E6, and E7 oncoproteins can activate the NF-κB pathway which is related to the progression of cervical carcinoma (208). NF-κB is the transcription factor to connect the immune system activation, chronic inflammatory responses, and carcinogenesis (209). Recent research suggested that the expression of E6/E7 was increased in cervical inflammation (205). Therefore, regulation of the translation and post-translation modification of these oncoproteins is essential to controlling the progression of inflammation. E6 and E7 mainly participated in the epigenetic regulations in host cells. E6 inhibited the activity of p300/CBP to affect the H3 acetylation, while E7 binds to p300/CBP to stimulate their activity (44, 210). The interactions of E6 and E7 with HATs downregulated the expression of the chemotactic interleukin 8 (IL-8) in immune cells, thereby affecting the inflammatory response (47, 211). E6 can also target HAT TIP60 to reduce the acetylation of histone H4 (45). The expression levels of HDAC1 and HDAC2 were increased, but the mechanism was not clear, which may be associated with the E6/E7-dependent elevation of SIRT1 expression (46).

Besides the acetylation, HPV infection can affect histone methylation distinctly. Changing the repressive mark H3K27me is a common histone modification in many cancers. HPV E6 and E7 stimulated the expression of FOXM1 and E2F1, respectively; both of them could bind the EZH2 promoter to enhance transcription (212). Otherwise, E6 also enhanced EZH2 transcription through inhibiting repression protein p53 (47). PRC1 bound to H3K27me-marked chromatin to silence gene expression through monoubiquitinating lysine 119 of histone H2A (213). However, researchers found that the H3K27me3 level was decreased in E6/E7-expressing cells. One reason is that E6/E7 upregulated the expression of KDM6A and KDM6B, which are demethylases targeting H3K27me (214). Controlling the balance of H3K27me may be a therapeutic intervention for HPV-associated malignancy.

#### Hepatitis Virus

HBV is hepatitis B virus with a single strand of DNA, 3.2 kb in size, the smallest among human DNA viruses. In the nucleus of infected cells, HBV formed a covalently closed circular DNA (cccDNA) minichromosome with host histone and non-histone proteins (196). HBV infection can cause acute, chronic, or occult hepatitis. With the infection, many people were at the risk of hepatocellular carcinoma (HCC). The main reason of treatment failure is the inability to eliminate the cccDNA (51). Strong evidence suggested that epigenetic regulations on both cccDNA and host genome were essential for viral life and pathogenesis.

HBx, bound to cccDNA, is the only regulatory protein encoded by HBV, playing an important role in the viral replication (215). HBx recruited p300/CBP acetyltransferase to cccDNA resulting in acetylation of H3 and H4, consequently activating transcription (49). HBx can directly interact with HADC1 and SIRT1 protein associated with the low HBV replication (50). In HBx mutant cells, the cccDNA-bound histones were hypoacetylated, resulting in low transcription (51). As mentioned above, HBx participated in the regulation of gene expression and viral replication through mediating histone acetylation.

Apart from histone acetylation, HBx also mediated methylation through affecting the level of H3K4me and H3K9me. In the absence of HBx, H3ac and H3K4me3 were decreased, and H3 dimethylation and tri-methylation (H3K9me) were increased with the concomitant transcriptional silencing and chromatin condensing. HBx affects histone methylation by several pathways. HBx stimulated the expression of SETDB1, the histone lysine 9-specific methyltransferase (51). HBx upregulated EZH2 expression and increased the half-life of EZH2. Furthermore, HBx increased the expression of the H3K4-specific methyltransferase, Set and MYND-domain containing 3 (SMYD3) (52). Histone methylation was correlated with the condensation of chromatin. In other words, HBx could regulate chromatin activation through mediating histone methylation (216).

HDV is hepatitis delta virus with a subviral satellite RNA virus that replicates only when it is surrounded by the helper protein-HBV surface antigen (217). Although the role of HDV in the development of hepatocellular carcinoma has not been well investigated, many epidemiological studies favored that it enhanced the development of liver cirrhosis and increased the risk of HCC with HBV superinfection. With the expression of HDV antigen HDAg, the transcription of CLU increased, which was associated with increased acetylation of histone H3 (53).

HCV is hepatitis C virus with a single-stranded RNA virus and is a unique human oncovirus that replicates in the cytoplasm exclusively (218). HCV infection induced the inhibition of histone H4 methylation/acetylation and histone H2AX phosphorylation through overexpression of phosphatase A catalytic subunit alpha (PP2Ac) with a significant expression change in genes important for hepatocarcinogenesis (54). Otherwise, histone H3 acetylation on lysine9/27, H3 acetylation on lysine14, and histone H2A acetylation on lysine5 also changed in HCV-infected cells (219).

For the role of histone methylation in HCV-infected cells, it has been reported that the expressions of KDM5B/JARID1B and LSD1, members of histone demethylase, were increased. Overexpression of KDM5B and LSD1 resulted in poor prognosis in HCC. Further studies may demonstrate KDM5B/LSD1 as a therapeutic target (55). The histone lysine methylase, G9a for lysine 9 of histone 3 (H3K9), is also associated with the progression of HCC prognosis (56). In China, overexpression of EZH2 was considered as a promising biomarker for HCC patients (220).

#### Human Immunodeficiency Virus

HIV-1 is human immunodeficiency virus type 1, with a single-stranded positive-sense RNA that replicated in CD4+ human immune cells. HIV-1 was integrated into the host cell genome after infection, establishing a stable latency which is the major obstacle for HIV cure (221). The proviral HIV genome was regulated by host epigenetic modification machinery, while HIV proteins affect the gene expression of host cells. The cellular epigenetic regulator mainly affected the viral promoter located in the 5′ long terminal repeat (LTR) sequence (222).

Histone deacetylases (HDAC1/2) were recruited on HIV LTRs; as a consequence, the transcription was suppressed (223). Histone acetylation was generally considered to promote gene expression, and the methylation on histones produced a complex scenario to control transcription. In microglial cells, CTIP2/BCL11B recruited many types of enzyme-chromatin-modifying complexes to establish the heterochromatic environment to repress HIV-1 gene expression. CTIP2 and LSD1 bound to the Sp1 site in LTR. CTIP2 sequentially recruited HDAC1/2 to acetylate H3 and HMT SUV39H1 to catalyze H3K9me3 which was recognized by HP1. In parallel, LSD1 recruited the COMPASS complex containing the histone methyltransferase SET1 to stimulate H3K4me3 (57, 224, 225). The bromodomain (BD) and extra-terminal domain (ET) protein, BRD4, consists of two conserved BDs that selectively bind to acetyl-lysine residues of histones. BRD4 was recruited to the HIV promoter to suppress gene expression, resulting in promotion of latency (58). EZH2 together with the EZH2-mediated H3K27me3 showed a higher level at the LTR of silenced HIV proviruses. Another methyltransferase G9a for histone H3 lysine 9 (H3K9) was responsible for transcriptional repression through promoting repressive demethylation at H3K9 (59). The relationship between PTM of histones and HIV-1 viral latency provides the potential therapeutic strategy. A successful HIV curative strategy needs to reverse HIV latency to purge hidden viral reservoirs or enhance HIV latency to silence HIV transcription permanently. Some epigenetic modifying agents have been suggested for transcription control of HIV-1 latency, such as histone deacetylase inhibitors (HDACi), histone methyltransferase inhibitors (HMTi), and histone demethylase inhibitors (226).

#### COVID-19

The coronavirus disease 2019 (COVID-19) pandemic was caused by acute respiratory syndrome coronavirus 2. The virus infection resulted in dysregulated immune responses and mass of acute inflammation. However, there is no specific drug. The virus life cycle and the host immune response to infection were associated with various epigenetic regulations, especially histone modifications which can be the therapeutic target. In a recent protein interactome analysis, hundreds of human proteins were identified to interact with SARS-CoV-2 proteins. Among these, eight proteins were associated with epigenetic regulations. HADC2 was identified to interact with NSP5 (non-structural protein 5) which participates in the formation of the replicase–transcriptase complex (60). HDAC2 can suppress inflammatory gene expression while the activity and expression of HDAC2 were inhibited in peripheral lung and alveolar macrophages with pulmonary diseases, such as COPD (227). The detection indicated that NSP5 was likely to inhibit HADC2, which then influenced HADC2-based inflammation responses. Another evidence was that ACE2 (angiotensin-converting enzyme 2) which was regulated by some histone modification proteins, such as HAT1 and HDAC2, was highly expressed in a number of severe COVID-19 patients (61, 228). SIRT1, another ACE2 epigenetic regulator, was also upregulated in several COVID-19 patients. Furthermore, ACE2 was especially regulated by histone methylation (H3K4mel and H3K4me3) and histone acetylation (H3K27ac) (62, 228).

The complication risks of COVID-19 are highly age-dependent. The age-dependent epigenetic regulation may be the foundation of age-associated severity of COVID-19 symptoms (62, 229). Histone modifications and the levels of histone proteins changed during aging, which dramatically influence chromatin compaction and gene expression. Solid evidence suggested that the acetylation level of H4 was reduced during aging. In kidney and liver tissue with age, the level of H4K20me3, a marker of constitutive heterochromatin, was increased. In some studies *in vitro*, the level of H3K9me3 was decreased (230). It is necessary to elucidate whether the epigenetic change during aging affects the severity of COVID-19 directly.

NETs were elevated in COVID-19 patients due to the higher level of 3 markers, cell-free DNA, myeloperoxidase-DNA, and citrullinated histone H3 (Cit-H3) (62, 63). Cit-H3 was one of the histone modifications, with the conversion of arginine to citrulline which is targeted by peptidylarginine deiminase 4 (PAD4) (64). Higher arginine contents change the charge distribution then affect the interaction with DNA, leading to chromatin decondensation and transcription activation (230). Meanwhile, Cit-H3 is an important epigenetic modification for stem cell pluripotency. Therefore, the modification of Cit-H3 might be crucial for epigenetic therapy of COVID-19.

## Concluding Remarks

Over the past two decades, the understanding of histone modifications in the pathogenesis of inflammatory diseases has been largely extended. In this review, we present an illustrative but not comprehensive overview of the key role of histone modifications in regulating the progression of inflammatory diseases. In this regard, we showed involvements of histone PTMs in AS, T2D, AD, psoriasis, asthma, COPD, CF-lung disease, inflammatory bowel disease, and virus-associated diseases, but it is not limited to the diseases mentioned here; there are still more inflammatory diseases related to histone modifications, such as periodontitis, spondyloarthritis, many types of cardiometabolic diseases, chronic kidney disease, colorectal cancer, and most common neurodegenerative diseases as well as demyelinating disorders. Numerous studies focusing on histone PTM-related molecular mechanisms using multi-omics methods combined with cell-based systems and experimental animal models have profoundly contributed to the current knowledge on this topic.

Accumulating data have shown that distinct histone modifications are enriched in inflammatory organisms suffering from chronic inflammatory diseases or virus-associated inflammatory diseases. The imbalance of activating and repressing histone modifications stimulates the development of a wide range of diseases ranging from autoimmunity, cardiovascular pathology, viscera injury, neurodegenerative disorder to cancer.

On the basis of salient cognition of these mechanisms, therapeutic applications of the histone modifications targeting for inflammatory diseases are expected to expand: HDAC inhibitors as clinical drugs; new generations of vaccines that alter histone PTMs levels; enzymes modulating histone PTMs; and development of inducers of distinct histone proteins for the treatment of inflammation paralysis in inflammatory diseases. In fact, epigenetic drugs nowadays attract particular interest to the clinic owing to their characteristic reversible and transient effects. Only continuous research on the mechanisms of histone modifications will be able to achieve these aims and fulfill the potentials through the comprehending of the role of histones post-translational modifications in inflammatory diseases.

## Author Contributions

YL, TQ, and XC conceived of the review and participated in its design. YL, TQ, GW, YQ, WW, YK, and TX summarized all the data and information acquired for the review. YL, TQ, TX and XC wrote the manuscript and prepared the table and figures. All authors contributed to the article and approved the submitted version.

## Funding

This study was supported by the National Natural Science Foundation of China (32100620, 32000363) and The Natural Science Foundation of Zhejiang Province (LQ21C060006).

## Conflict of Interest

The authors declare that the research was conducted in the absence of any commercial or financial relationships that could be construed as a potential conflict of interest.

## Publisher’s Note

All claims expressed in this article are solely those of the authors and do not necessarily represent those of their affiliated organizations, or those of the publisher, the editors and the reviewers. Any product that may be evaluated in this article, or claim that may be made by its manufacturer, is not guaranteed or endorsed by the publisher.
